# An American Text-Book of Gynecology

**Published:** 1895-03

**Authors:** 


*An American Text-Book of Gynecology.* Edited by J. M. BALDY, M.D. Pp. 713. Philadelphia: W. B. SAUNDERS. London: F. J. REBMAN. 1894.

The method of combined authorship by which this book is written seems to give most excellent results; for by this means we not only obtain such sound and well-tried principles and practice as the co-workers feel that they can collectively recommend, but we also avoid errors which may arise from individual bias.

The work has been carefully prepared, and the subject is treated in a clear and readable manner. An important feature is the completeness with which the text has been supplemented and enforced by means of illustrations. One has only to turn to the plates showing the various steps by which the operation of hysterectomy—by clamp or ligature—is performed to see how extremely helpful such illustrations are, and how well the authors have done this part of their work. We feel, however, that here and there the application of photography has been somewhat abused: for we do not think it necessary to have two pictures of a faulty gynæcological position, as well as one of the correct attitude; and again, if we must have a picture of an operator in correct costume bathing his hands in permanganate of potash, surely we do not require to see the same gentleman washing in oxalic acid afterwards. If the clothing in these latter pictures is meant to compensate for the absence of garments in the former ones we have referred to, we should perhaps have less cause for complaint.

On the whole, we are very pleased with the work, which is well up to date. We are particularly impressed with the chapter dealing with genital tuberculosis and uterine neoplasms. It is worth noting that the authors regard the use of ergot with more favour than electricity in the treatment of uterine fibroids.

The book is very free from typographical errors, but the "Right labia majora," on p. 182, should obviously be "Right labium majus." The index is very complete, the book is well printed, and the illustrations most artistically executed; but some of the half-tone pictures do not compare favourably with the photographic and coloured plates.

